# Virtual and Augmented Reality in Cardiac Surgery

**DOI:** 10.21470/1678-9741-2020-0511

**Published:** 2022

**Authors:** Arian Arjomandi Rad, Robert Vardanyan, Aleksandra Lopuszko, Christina Alt, Ingo Stoffels, Bastian Schmack, Arjang Ruhparwar, Konstantin Zhigalov, Alina Zubarevich, Alexander Weymann

**Affiliations:** 1 Faculty of Medicine, Imperial College London, London, United Kingdom.; 2 Department of Thoracic and Cardiovascular Surgery, West German Heart and Vascular Center, University of Duisburg-Essen, Essen, Germany.; 3 Department of Dermatology, University of Duisburg-Essen, Essen, Germany.; 4 Faculty of Medicine, Barts and The London School of Medicine and Dentistry, London, United Kingdom.

**Keywords:** Virtual Reality, Augmented Reality, Cardiac Surgical Procedures, Technology, Medicine

## Abstract

Virtual and augmented reality can be defined as a three-dimensional real-world simulation allowing the user to directly interact with it. Throughout the years, virtual reality has gained great popularity in medicine and is currently being adopted for a wide range of purposes. Due to its dynamic anatomical nature, permanent drive towards decreasing invasiveness, and strive for innovation, cardiac surgery depicts itself as a unique environment for virtual reality. Despite substantial research limitations in cardiac surgery, the current literature has shown great applicability of this technology, and promising opportunities.

**Table:** 

Abbreviations, acronyms & symbols	
3D	= Three-dimensional
AR	= Augmented reality
CT	= Computed tomography
Cx	= Circumflex
LAD	= Left anterior descending artery
LIMA	= Left internal mammary arteries
MICS	= Minimally invasive cardiac surgery
RCA	= Right coronary artery
VR	= Virtual reality

## INTRODUCTION

**Fig. 1:**
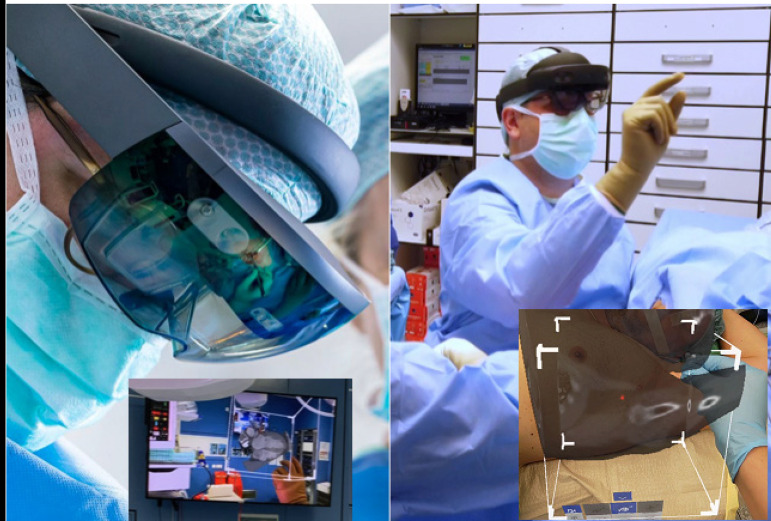
Practical implementation of the virtual reality in the surgical operating room.

**Fig. 2:**
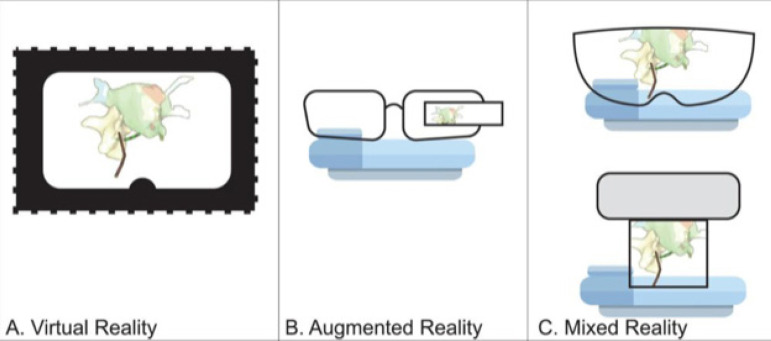
Schematic display of extended realities. A) Virtual reality: digital space is completely separated from the environment. B) Augmented reality: digital space is integrated into the users’ natural environment. C) Mixed reality: the user is able to stay in his natural environment and still interact with the digital information.

Virtual and augmented reality (VR and AR) can be defined as a three-dimensional (3D) real-world simulation allowing the user to directly interact with it (Figure 1) ^[1]^. Through the integration of imaging data and input from users ^[2]^, VR delivers a 3D graphical output which can be then visualized through a wearable headset (Figure 2). Throughout the years, VR has gained great popularity in medicine and is currently being adopted for a wide range of purposes including medical education, stroke rehabilitation, and teaching of surgical techniques, particularly laparoscopy ^[3,4]^. Despite its great advances in numerous areas of medicine, the future potential innovative impact of VR in cardiac surgery has not been extensively discussed yet, with no formal integration of this technology in this specialty. Nevertheless, due to its dynamic anatomical nature, permanent drive towards decreasing invasiveness, and strive for innovation, cardiac surgery depicts itself as a unique environment for VR.

## COMMENTS

### The Role of VR in Undergraduate and Postgraduate Cardiac Teaching

See one, do one, teach one - the method of “learning-by-doing” paired with “on-the-job-training” is one of the most popular surgical training methods. Ever since the surgeons have been using real-life scenario simulations, practical skills sessions, and video sessions ^[5]^ as means of improving their dexterity, the desire for more precise and even more stimulating methods has been growing. As the works to develop robots and interactive technologies to support surgical performance have been underway ^[6]^, the VR and active engagement techniques came along. Initially, they have been thought to allow 3D view and better insight into the anatomy and procedures. Since the introduction of 3D visualisation, many alternative ways to utilize the new techniques have been developed. The real-time interaction, decreasing invasiveness of procedures, and a reliable tool of surgical skills assessments are only a few to mention for a variety of opportunities given by the VR training in medicine and surgery.

### The Role of VR in Preoperative, Intraoperative, and Postoperative Cardiac Surgery

**Fig. 3:**
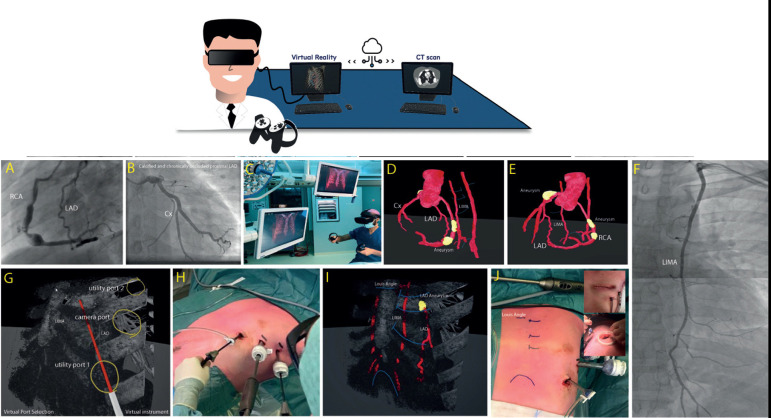
Augmented reality in cardiac surgery (by Sadeghi et al. ^[14]^). A and B) Coronary angiography with proximally calcified aneurysm and an occlusion of the left anterior descending artery (LAD) with collateral retrograde filling from the right coronary artery (RCA) and no abnormalities in the left circumflex (Cx) artery. C) Reconstructions of a computed tomography (CT) scan were made by rendering three-dimensional virtual reality (VR) images. D and E) Reconstruction of the CT scan. G-J) Immersive VR was used to plan for the insertion location of thoracoscopic ports (for left internal mammary arteries [LIMA] harvesting) and for determining the ideal location for anterior mini-thoracotomy.

Perhaps one of the greatest potential future applications of VR systems in cardiac surgery will be their assistance and support for the much-desired shift from open sternotomy procedures to minimally invasive ones. Reducing patient’s intraoperative trauma and allowing for faster postoperative recovery have been some of the priorities of cardiac surgery over the past decades, thus leading to the development of endoscopically- and robotically-assisted minimally invasive cardiac procedures ^[7]^. Nevertheless, the shift towards minimally invasive cardiac surgery (MICS) has been taking place at a slow pace due to numerous limitations. The latter include its learning curve and the shortage of safe and structured training methods, the difficulties in port location in order to enable effective X-ray and angiography coverage, and the limited access to anatomical and surgical targets ^[8-12]^. Indeed, even at wide-angled panoramic views, the use of an endoscope for the visualisation of complex anatomical structures in a 3D and dynamic environment proves to be complicated and could be considered a limitation to MICS. In light of these limitations, VR might be able to offer unique opportunities to improve the visualisation of surgical targets and enhance the beating-heart intracardiac surgical outcomes (Figure 3) ^[13,14]^.

### Is VR and AR Implementation in Cardiac Surgery Cost-Effective?

Since the conception of VR and AR in a healthcare setting, the cost of its implementation has been a significant barrier to its use in surgery. This is particularly true for cardiac surgery which requires VR and AR simulations to be of high-fidelity and precision, regardless of the purpose of the simulation. For this reason, the sheer processing power alone of a computer suitable for such VR simulations rendered the technology poorly cost-effective ^[15]^. However, over the past decade, the economic barriers to the use of VR and AR in a surgical setting have diminished as cheaper technology with significantly stronger processing power becomes commercialized and the opportunities of VR to improve patient safety, surgical training, and audit quality becomes evident.

In particular, implementation costs for VR and AR technology in a surgical setting are becoming more affordable through the use and adaption of commercially available hardware that is non-specific in its use, such as in the case of recently developed VR headsets that provide high-quality visuals and realistic surgeon hand interactions ^[16]^. It would not be unreasonable to assume that potentially significant advances in reducing costs when simulating cardiothoracic surgery can be made, particularly comparing to almost a decade ago.

Moreover, VR has shown promising and potential cost-effectiveness in presurgical and interventional planning in congenital cardiac surgery. Whilst in the short-term, VR incurs significant initial set-up and implementation costs relative to 3D printing heart models, in the long-term it has been suggested to control costs, reduce material wastage, and allow for a more immersive and detailed experience for the multidisciplinary team by allowing improved depth perception and visualization ^[17]^. In fact, it can also be argued that relatively similar start-up costs exist for 3D printing heart models, including the recruitment of appropriate technicians and specialists to facilitate the operation ^[18]^. However, beyond the initial fixed set-up costs, VR may reduce costs compared with 3D printing, as the need for regular purchases of materials and disposal of plastic waste is removed ^[18]^.

Within surgical teaching, VR can eliminate the even greater costs of cadaveric and animal tissue models whilst providing a wider range of anatomical variation ^[19]^. Furthermore, VR allows for repetitions of the learning experience, which not only improves the efficacy of the curricula, but it can also lead to further cost savings in the long-term. Particularly in regard to robotic surgery, which is associated with significant costs in its use and implementation, VR has been shown to be a valuable alternative to operating room learning sessions.

## CONCLUSION

From our perspective, VR and AR brings opportunities to rapidly develop the field of cardiac surgery. VR has gained growing popularity and adoption in different medical and surgical fields, being embedded from medical education to preoperative planning, operative assistance, and even postoperative support to patients. The drive for innovation in cardiac surgery has been growing over the past years in search of methods to maximize patient outcomes and quality of life and improve training pathways for young surgeons. Although substantial research limitations persist in the field of VR and AR application to cardiac surgery, the current literature has shown great applicability of this technology, and promising opportunities.

**Table:** 

Authors' roles & responsibilities	
AAR	Substantial contributions to the conception of the work; agreement to be accountable for all aspects of the work in ensuring that questions related to the accuracy or integrity of any part of the work are appropriately investigated and resolved; final approval of the version to be published
RV	Substantial contributions to the conception of the work; agreement to be accountable for all aspects of the work in ensuring that questions related to the accuracy or integrity of any part of the work are appropriately investigated and resolved; final approval of the version to be published
AL	Substantial contributions to the conception of the work; agreement to be accountable for all aspects of the work in ensuring that questions related to the accuracy or integrity of any part of the work are appropriately investigated and resolved; final approval of the version to be published
CA	Substantial contributions to the conception of the work; agreement to be accountable for all aspects of the work in ensuring that questions related to the accuracy or integrity of any part of the work are appropriately investigated and resolved; final approval of the version to be published
IS	Substantial contributions to the conception of the work; agreement to be accountable for all aspects of the work in ensuring that questions related to the accuracy or integrity of any part of the work are appropriately investigated and resolved; final approval of the version to be published
BS	Substantial contributions to the conception of the work; agreement to be accountable for all aspects of the work in ensuring that questions related to the accuracy or integrity of any part of the work are appropriately investigated and resolved; final approval of the version to be published
AR	Substantial contributions to the conception of the work; agreement to be accountable for all aspects of the work in ensuring that questions related to the accuracy or integrity of any part of the work are appropriately investigated and resolved; final approval of the version to be published
KZ	Substantial contributions to the conception of the work; agreement to be accountable for all aspects of the work in ensuring that questions related to the accuracy or integrity of any part of the work are appropriately investigated and resolved; final approval of the version to be published
AZ	Substantial contributions to the conception of the work; agreement to be accountable for all aspects of the work in ensuring that questions related to the accuracy or integrity of any part of the work are appropriately investigated and resolved; final approval of the version to be published
AW	Substantial contributions to the conception of the work; agreement to be accountable for all aspects of the work in ensuring that questions related to the accuracy or integrity of any part of the work are appropriately investigated and resolved; final approval of the version to be published
